# Mixed infection, risk projection, and misdirection: Interactions among pathogens alter links between host resources and disease

**DOI:** 10.1002/ece3.7781

**Published:** 2021-06-22

**Authors:** Alexander T. Strauss, Lucas Bowerman, Anita Porath‐Krause, Eric W. Seabloom, Elizabeth T. Borer

**Affiliations:** ^1^ Department of Ecology, Evolution, and Behavior University of Minnesota St. Paul MN USA; ^2^ Odum School of Ecology University of Georgia Athens GA USA

**Keywords:** barley/cereal yellow dwarf, coinfection, community ecology, competition, disease, facilitation, nutrient, virus

## Abstract

A growing body of literature links resources of hosts to their risk of infectious disease. Yet most hosts encounter multiple pathogens, and projections of disease risk based on resource availability could be fundamentally wrong if they do not account for interactions among pathogens within hosts. Here, we measured infection risk of grass hosts (*Avena* *sativa*) exposed to three naturally co‐occurring viruses either singly or jointly (barley and cereal yellow dwarf viruses [B/CYDVs]: CYDV‐RPV, BYDV‐PAV, and BYDV‐SGV) along experimental gradients of nitrogen and phosphorus supply. We asked whether disease risk (i.e., infection prevalence) differed in single versus co‐inoculations, and whether these differences varied with rates and ratios of nitrogen and phosphorus supply. In single inoculations, the viruses did not respond strongly to nitrogen or phosphorus. However, in co‐inoculations, we detected illustrative cases of 1) resource‐dependent antagonism (lower prevalence of RPV with increasing N; possibly due to competition), 2) resource‐dependent facilitation (higher prevalence of SGV with decreasing N:P; possibly due to immunosuppression), and 3) weak or no interactions within hosts (for PAV). Together, these within‐host interactions created emergent patterns for co‐inoculated hosts, with both infection prevalence and viral richness increasing with the combination of low nitrogen and high phosphorus supply. We demonstrate that knowledge of multiple pathogens is essential for predicting disease risk from host resources and that projections of risk that fail to acknowledge resource‐dependent interactions within hosts could be qualitatively wrong. Expansions of theory from community ecology theory may help anticipate such relationships by linking host resources to diverse pathogen communities.

## Graphical abstract

When hosts encounter multiple pathogens, their risk of infection can depend on the nutrients available to the host. We experimentally show that interactions among pathogens fundamentally transform relationships between host resources and disease. Emergent patterns of infection risk arise from both resource‐dependent antagonism and resource‐dependent facilitation within hosts.

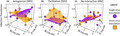

## 1 INTRODUCTION

All species—including plants, animals, and humans—consume resources and encounter pathogens. Consequently, a growing body of literature seeks to link variation in host resources to their risk of infectious disease (Becker et al., 2018; Dordas, 2008; Hite et al., 2019; Smith, 2007; Veresoglou et al., 2013). Resources can lower infection risk, for example, by boosting immune defenses of hosts (Becker et al., 2018; Pedersen & Greives, 2008). Then again, resources can elevate infection risk by fueling the growth of pathogens inside hosts (Clasen & Elser, 2007; Frost et al., 2008; Whitaker et al., 2015), increasing the likelihood of successful, systemic infection (Mur et al., 2017). Importantly, anthropogenic changes are altering the availability of resources to plants via fertilization and environmental eutrophication, animals via anthropogenic subsidies, and humans via altered food production and diet. As these global changes intensify, it is becoming increasingly urgent to understand how these changes in host resources shape disease in plant (Huber & Haneklaus, 2007; Mur et al., 2017; Veresoglou et al., 2013), animal (Becker et al., 2018; Hite et al., 2019), and human hosts (Prentice et al., 2008; Rohr et al., 2019).

Although most research linking resources to disease focuses on single pathogens in isolation, these relationships may fundamentally change when hosts face multiple pathogen species or strains. Such mixed infections are ubiquitous in nature and important because they can alter host immune responses, disease symptoms, and pathogen evolution (Rynkiewicz et al., 2015; Seabloom et al., 2015; Tollenaere et al., 2015). Relationships between resources and infection risk can differ in mixed infections, because different pathogens, including viruses (Kendig et al., 2020; Lacroix et al., 2017), can be limited by different resources (Seabloom et al., 2013; Smith & Holt, 1996; Wale et al., 2017). Additionally, pathogens often interact inside hosts, either antagonistically (e.g., via competition) or synergistically (e.g., via immunosuppression; Abdullah et al., 2017; DaPalma et al., 2010; Karvonen et al., 2011; Pedersen & Fenton, 2007). Critically, the strength of these interactions also can depend on resources (Box 1; van Lettow et al., 2003; Lacroix et al., 2014; Lange et al., 2014; Budischak et al., 2015; Wale et al., 2017; Kendig et al., 2020). Thus, focusing on single pathogens in isolation could mislead predictions for how resources shape infection risk when hosts face more realistic and diverse pathogen communities. For example, projections could *overestimate* disease risk if certain resource conditions strengthen antagonistic interactions among pathogens. On the other hand, projections could *underestimate* disease risk if facilitation among pathogens increased in certain nutritional environments.

BOX 1 **How can host resources mediate interactions among pathogens?**

**Table:** 

When multiple pathogens co‐occur in a host, they show one of three classes of interactions: antagonism, facilitation, or weak to no interactions at all. Each class of interaction could arise from a variety of mechanisms, and each mechanism could hinge upon host resources. **Antagonism**: *Presence of one pathogen decreases the likelihood of successful infection by another*. Mechanisms for antagonistic interactions among pathogens include competition for resources, competition for space, and apparent competition mediated by the host immune system (cross‐protection). Host resources could mediate these interactions if they limit pathogen growth rate, the host immune system, or host size (Lacroix et al., 2014; Lange et al., 2014; Wale et al., 2017). **Facilitation**: *Presence of one pathogen increases the likelihood of successful infection by another*. Mechanisms for facilitation among pathogens include immunosuppression, immune distraction, mechanical facilitation (i.e., overcoming host physical defenses), and, for closely related viruses, heterologous encapsidation. Host resources could mediate these interactions by fueling host immune function, physical defenses, or pathogen growth rate (Budischak et al., 2015; Kendig et al., 2020; van Lettow et al., 2003). **Weak or no interaction:** *Presence of one pathogen does not affect the likelihood of successful infection by another*. Of course, pathogens need not interact within a host, for example, if they infect different tissues, are targeted by different components of the host immune system, or if infection depends more on external factors (e.g., vectors or other means of dispersal/transmission) than factors internal to the host.

Here, we experimentally ask whether exposure to multiple pathogens alters relationships between resources and disease risk using a naturally co‐occurring community of plant viruses. We hypothesized that rates of infection by three viruses in isolation would differ with nitrogen (N) or phosphorus (P) supply and that the strength of interactions among the viruses would also vary with host resources, as suggested by laboratory experiments (Kendig et al., 2020; Lacroix et al., 2014), field experiments (Kendig et al., 2017; Seabloom et al., 2013), and field observations (Seabloom et al., 2010). We grew grass hosts (*Avena* *sativa*) under crossed gradients of N and P (three levels each) and exposed them to three species of barley and cereal yellow dwarf virus (CYDV‐RPV, BYDV‐SGV, and BYDV‐PAV), either singly or jointly. We found that the viruses did not respond strongly to N or P in single inoculations, but that risk (i.e., infection prevalence) for co‐inoculated hosts was highest at combinations of low N and high P (low N:P ratio). This pattern emerged from resource‐dependent interactions within hosts (Box 1). Among the three viruses, we detected illustrative cases of 1) resource‐dependent antagonism (for RPV, especially with higher N), 2) resource‐dependent facilitation (for SGV, especially with lower N:P), and 3) no difference between single and co‐inoculations (for PAV). These outcomes suggest several applications of theory from community ecology to disease, including resource ratio and metacommunity theory (Rynkiewicz et al., 2015; Smith & Holt, 1996; Strauss et al., 2019), and emphasize that predictions linking host resources to infection risk could be fundamentally wrong if they do not account for interactions among pathogens within hosts.

## 2 MATERIALS & METHODS

### 2.1 Study system

Barley and cereal yellow dwarf viruses (B/CYDVs) are an economically and ecologically important group of generalist RNA viruses that are capable of infecting over one hundred species of grasses (Irwin & Thresh, 1990). They are obligately transmitted among grasses by aphid vectors, with different species of aphid transmitting different species of virus. The aphid *Schizaphis graminum* transmits BYDV‐SGV, *Sitobion avenae* transmits BYDV‐PAV, and *Rhopalosiphum padi* transmits several viruses including CYDV‐RPV (Rochow, 1969; Seabloom et al., 2009). Hereafter, we refer to these viruses as SGV, PAV, and RPV, respectively. Coinfections among two or more viruses are common in natural plant communities (Seabloom et al., 2010) and agricultural settings (Rochow, 1979), especially for viruses that share vectors (Kendig et al., 2017). In a field study that tested for presence of five B/CYDVs (including SGV, PAV, and RPV), mean viral richness in infected hosts ranged from ~2–3 unique virus species (Seabloom et al., 2013). The number of viruses within a host is an important agricultural metric of disease, because coinfected plants exhibit more severe symptoms (Baltenberger et al., 1987).

B/CYDVs are well suited to study relationships between resource availability and infection risk, because they respond to variation in nitrogen and phosphorus supply to hosts (Rua et al., 2013; Seabloom et al., 2009). Some of the observed virus responses in the field (Kendig et al., 2017; Seabloom et al., 2010, 2013) could reflect effects of nutrients on aphid demography or behavior (Strauss et al., 2020). In contrast, responses in laboratory experiments with controlled aphid exposure can isolate effects of the resource environment of the host (i.e., tissue chemistry). For example, phosphorus decreased RPV prevalence, while nitrogen did not (Lacroix et al., 2014); nitrogen addition increased titer of PAV (Whitaker et al., 2015), but decreased titer of RPV (Lacroix et al., 2017).

These responses to nitrogen and phosphorus also depend on interactions among viruses, although previous experiments have only investigated pairwise interactions. B/CYDVs interact antagonistically through a variety of mechanisms (Power, 1996). First, they likely compete for shared resources, since key elements—such as P—are required for viral replication (Hall & Little, 2013). Competition can also be mediated by plant traits (e.g., Lacroix et al., 2017), and closely related B/CYDVs cross‐protect against one another via the host immune system (Wen et al., 1991). Facilitation among viruses is also possible (Kendig et al., 2020). B/CYDVs can hijack and replicate in the capsid proteins of heterospecifics (“heterologous encapsidation”) (Wen & Lister, 1991). They also can inhibit the RNA silencing defenses of hosts (Liu et al., 2012), and if one virus inhibits host immune function, it could facilitate infection by others. Any of these interactions within hosts could differ with nitrogen or phosphorus supply (Box 1). For example, co‐inoculation with PAV reduced risk of infection by RPV (Lacroix et al., 2014), and co‐inoculation with RPV *increased* titer of PAV (Kendig et al., 2020), but both effects only occurred at low levels of N and P. More complex interactions are likely to arise when hosts are exposed to more diverse viral communities.

It is important to note that nutrients can have differing effects on infection risk (prevalence; probability of infection after aphid exposure), viral titer (abundance of virions within a successfully, infected host), and likelihood of transmission out of the host (Kendig et al., 2020; Lacroix et al., 2017). All three responses reflect important biological processes for viruses in nature. The current experiment focuses on infection risk for hosts after controlled exposure via aphids. It is equally important to note that aphid species differ in their efficiency of transmitting their respective viruses (Rochow, 1969). Thus, while we control for many factors that could shape infection risk in nature (e.g., host conditions, environmental conditions, number of aphids, and duration of exposure), differences in aphid transmission efficiency are an inherent feature of this study system. Therefore, effects of resources and co‐inoculation are best interpreted as relative changes on infection risk, for each virus‐vector species combination.

### 2.2 Experimental setup & design

We measured infection risk for hosts in an experiment that manipulated the supply rate of two resources (three levels of N crossed by three levels of P; five unique N:P ratios) and richness of inoculated viruses (hosts exposed to RPV, SGV, and PAV, either singly or all together). All grass hosts were planted in individual pots (60 mm tall, 27 mm diameter, 55 ml per pot), isolated in mesh “bug dorms” (32.5 × 32.5 × 77 cm; 160 μm mesh; MegaView Science Co.), and grown in a climate‐controlled room (25℃; 18:6 light:dark; 2 × 40 W cool white fluorescent bulbs). We planted seeds (*Avena* *sativa*, cv Coast Black oat, National plant germplasm system, USDA, USA) in sterilized, water‐saturated, nutrient‐free media (70% medium vermiculite [Sun Gro Horticulture], 30% Turface MVP [Turface Athletic, Buffalo Grove] by volume). Thereafter, we watered each plant 5 ml twice per week with modified Hoagland's nutrient solution to create crossed exponential gradients of nitrogen and phosphorus supply (see appendix for details). Plants received one of three levels of nitrogen (7.5, 52.5, and 375 µM) and one of three levels of phosphorus (1, 7, and 50 µM), creating nine unique nutrient combinations of N and P and five unique N:P ratios. The highest and lowest levels of N and P are consistent with previous experiments and represent a reasonable range of natural (i.e., nonagricultural) conditions favorable for plant growth (Kendig et al., 2020; Lacroix et al., 2014, 2017).

When plants were two weeks old, we introduced viruses via aphid vectors. We reared colonies of nonviruliferous aphids (i.e., not yet carrying a virus) including *R*. *padi*, *S*. *graminum*, and *S*. *avena* using standard protocols (see appendix for details). Aphids used in the experiment acquired their respective viruses by feeding from plant tissue known to be infected with RPV, SGV, or PAV for 48‐hr viral acquisition access periods (Gray, 2008). Then, we transferred these newly viruliferous aphids to the experimental plants. We placed viruliferous aphids in a single mesh “sleeve” (2.5 × 8.5 cm, 118 μm; supported with a bamboo stick and sealed with Parafilm) that was attached to the oldest leaf of each plant. Plants in single virus treatments received two aphids each. Plants in the mixed virus treatments received two of each viruliferous aphid, plus two nonviruliferous *R*. *padi* due to a logistical error (8 aphids in total). Importantly, aphids that had acquired viruses from different infected leaves during the acquisition periods were distributed evenly among treatments to control for any variation in viral titer in the acquisition leaves. We replicated hosts 10x (single virus treatments) or 20× (mixed virus treatment; greater replication since more outcomes were possible) at each combination of N and P (450 plants total). We allowed the viruliferous aphids to feed and potentially transmit viruses for five days. After this controlled inoculation access period, we manually killed all aphids, removed the mesh sleeves from plants, and ensured that we had eliminated all aphids by applying pesticide and ladybug predators.

After the inoculation access period, we continued to supply plants with water and their respective nutrient treatments for three weeks before diagnosing infections. This three‐week period for viral growth—and potential competition—ensured that infections became systemic and easier to detect in plants that had become infected (Kendig et al., 2020). Then, we harvested each plant to diagnose infection(s) with standard laboratory procedures (Lacroix et al., 2014). In short, we extracted total RNA from leaf samples, synthesized cDNA using random hexamers, amplified any viral cDNA with primers specific to RPV, SGV, or PAV, and visualized PCR products with gel electrophoresis (see appendix for details).

### 2.3 Statistical analyses

All statistical analyses were conducted in R version 3.5.2 (R Core Team, 2017). We used logistic regressions (function: glm) to ask whether infection risk (synonymous with infection prevalence; proportion of hosts that became infected during the controlled inoculations) differed with nitrogen (N), phosphorus (P), richness of inoculated viruses (R), or any two‐way interactions (hereafter: *crossed NxP* *models*). Since our experimental design allowed us to distinguish between effects of N and P versus N:P ratio (nine combinations of N and P; five unique N:P ratios), we also fit models that tested for effects of N:P ratio (hereafter: *N:P* *ratio models*). Separate models tested risk of infection by each virus (RPV, SGV, and PAV). If interaction terms were not significant, we removed them to avoid overfitting (Table 1). Among the co‐inoculated hosts, we asked whether overall infection prevalence (proportion of hosts infected by one or more viruses) or realized viral richness (number of unique viruses successfully infecting a host) differed with N, P, their interaction, or their ratio (Table 2). For the realized richness response, we used generalized linear models with Poisson‐distributed errors that separately considered all co‐inoculated hosts or just the subset that became infected.

**TABLE 1:** Effects of nitrogen (N), phosphorus (P), and inoculated viral richness (R; either single or co‐inoculation) on infection prevalence of three viruses (barley/cereal yellow dwarf viruses [B/CYDV’s]: CYDV‐RPV, BYDV‐SGV, and BYDV‐PAV) in plant hosts (*Avena* *sativa*). Separate models consider N and P as crossed factors (top) or as a resource ratio (N:P; bottom). Significant effects from logistic regressions for each virus (columns) are bolded; interaction terms removed if not significant. Results are shown in Figure 2 & Figure S1; post hoc analyses separate single versus co‐inoculations (Table S3)

Model & terms	Response: RPV (Figure 2a; *antagonism*)			Response: SGV (Figure 2b; *facilitation*			Response: PAV (Figure 2c; *no interaction*)		
Crossed N × P	EST.	*S.E*.	*p*‐value	EST.	*S.E*.	*p*‐value	EST.	*S.E*.	*p*‐value
Intercept^a^	−0.11	0.48	.81	−1.21	0.56	.030	−2.28	0.46	<.0001
N^b^	0.28	0.18	.11	0.16	0.19	.41	**0.27**	**0.11**	.**019**
P^b^	0.19	0.25	.45	−0.03	0.20	.89	−0.03	0.11	.79
R^c^	**1.49**	**0.57**	.**009**	1.10	0.62	.076	0.02	0.37	.93
N × P	0.01	0.06	.92	**−0.14**	**0.06**	.**018**			
N × R	**−0.84**	**0.19**	**<.0001**	−0.36	0.21	.082			
P × R	−0.35	0.18	.053	**0.59**	**0.21**	.**005**			

N:P ratio	EST.	*S.E*.	*p‐*value	EST.	*S.E*.	*p‐*value	EST.	*S.E*.	*p*‐value
Intercept	1.05	0.30	.017	−1.62	0.38	<.0001	−2.08	0.36	<.0001
*N*:P^b^	**−0.12**	**0.06**	.**039**	0.11	0.12	.36	0.15	0.08	.062
R	**−0.79**	**0.28**	.**004**	**2.38**	**0.45**	**<.0001**	0.03	0.08	.93
*N*:P × R				**−0.47**	**0.14**	**<.001**			

^a^ Intercept in Crossed N × P model is log odds of single inoculations at lowest levels of N and P in the experiment.

^b^ N, P, and N:P ratio are log transformed to reduce statistical leverage.

^c^ R = inoculated viral richness.

**TABLE 2:** Effects of nitrogen (N) and phosphorus (P) on infection prevalence and viral richness (barley/cereal yellow dwarf viruses [B/CYDV’s]: CYDV‐RPV, BYDV‐SGV, and BYDV‐PAV) in co‐inoculated hosts (Figure 3). Separate models consider N and P as crossed factors (top) or as a resource ratio (N:P; bottom). Significant effects from linear models are bolded; interaction terms removed if not significant

Model & terms	Response: Prevalence of infection by one or more viruses (Figure 1e & Figure 3a)			Response: Viral richness across all hosts (Figure 3b)			Response: Viral richness of infected hosts (Figure 3c)		
Crossed N×P	EST.	*S.E*.	*p‐*value	EST.	*S.E*.	*p‐*value	EST.	*S.E*.	*p‐*value
Intercept^a^	1.62	0.52	.002	0.19	0.17	.25	0.46	0.14	<.001
N^b^	−0.24	0.19	.20	−0.05	0.07	.49	−0.05	0.05	.26
P^b^	0.50	0.27	.063	0.11	0.06	.083	0.011	0.04	.80
N×P	**−0.18**	**0.09**	.**044**	**−0.06**	**0.03**	.**046**			

N:P ratio	EST.	*S.E*.	*p*‐value	EST.	*S.E*.	*p‐*value	EST.	*S.E*.	*p‐*value
Intercept	1.84	0.30	<.0001	0.30	0.09	<.001	0.45	0.09	<.0001
*N*:P^b^	**−0.27**	**0.09**	.**0018**	**−0.09**	**0.03**	.**0038**	−0.03	0.03	.32

^a^ Intercept in Crossed *N* x P models is log odds at lowest levels of N and P in the experiment.

^b^ N, P, and N:P ratio are log transformed to reduce statistical leverage.

For all analyses, we log transformed supply rates of N and P as well as N:P ratio to create evenly distributed predictor variables and reduce statistical leverage. For the crossed NxP models, we shifted N so that the intercept term would reflect log odds of infection at the lowest levels of N and P in the experiment. We followed models that yielded significant interactions with post hoc analyses, separately for single versus co‐inoculations, to better interpret effects of N and P (Table S3). To check whether our unbalanced design (lower replication for single [10×] than co‐inoculations [20×]) influenced the interpretation of our results, we re‐ran models the models for single inoculations with artificially inflated sample sizes (20×; Table S3). This analysis showed that—if anything—we were underestimating the strength of interactions among the viruses (see appendix for details). Finally, we also fit models that pooled all single inoculations together, both with and without “virus species” as a factor, which highlighted differences in transmission efficiency of the different aphid vectors (Table S4).

**FIGURE 1:**
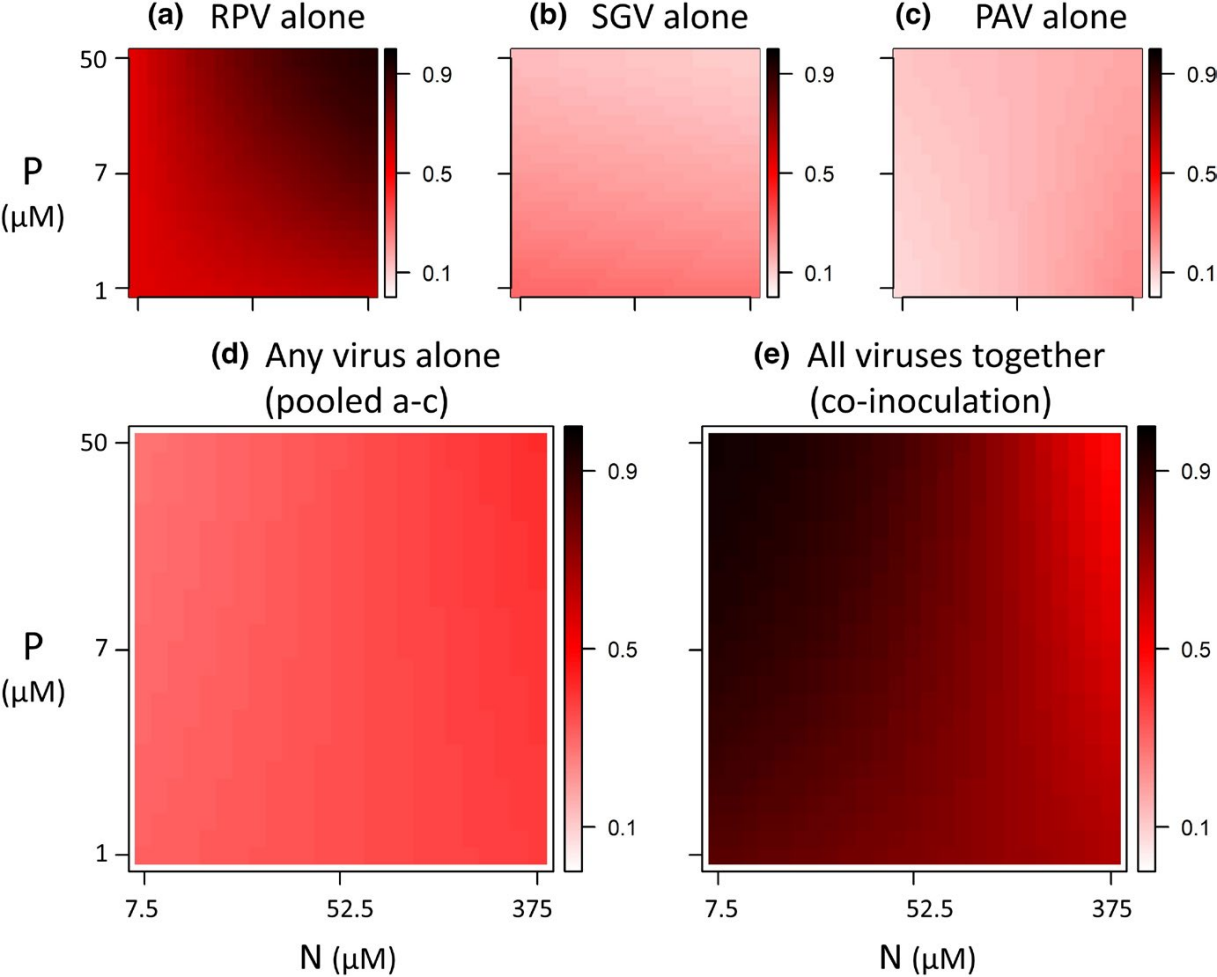
Heat maps project infection risk across gradients of nitrogen and phosphorus supply. Hosts (oats, Avena sativa) are exposed to one or three viruses (barley/cereal yellow dwarf viruses [B/CYDV’s]: CYDV‐RPV, BYDV‐SGV, and BYDV‐PAV) along gradients of nitrogen and phosphorus supply (three levels each). Colors show impacts of resource supply on infection risk (i.e., infection prevalence; the proportion of exposed hosts that became infected), as fitted by logistic regression models. The risk of infection by each virus alone (single inoculations: A‐D) qualitatively misdiagnoses the risk of infection when hosts are inoculated with all three viruses together (co‐inoculations: E). (a) Risk of infection by RPV alone is higher than (b) SGV or (c) PAV, but none of the viruses respond significantly to N, P, or N:P ratio in isolation (although sample size is admittedly low—see Table S3). (d) When pooling the single inoculations (ignoring differences among viruses), infection risk for hosts does not vary with nutrients. (e) However, infection risk for hosts co‐inoculated with all three viruses together is significantly higher under conditions of low N and high P (high N:P ratio). This emergent pattern arises from resource‐dependent interactions among viruses within hosts (Box 1; Figure 2)

**FIGURE 2:**
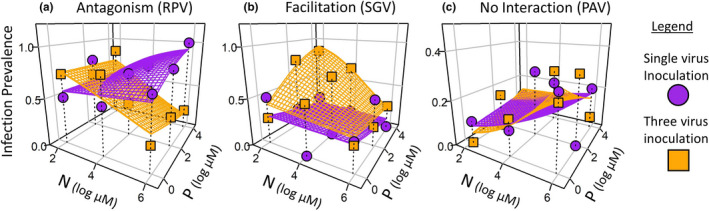
Infection prevalence of three viruses illustrates three types of resource‐dependent outcomes within hosts: antagonism, facilitation, and weak or no interaction. Hosts (oats, Avena sativa) are grown under combinations of nitrogen and phosphorus supply (three levels each) and inoculated with three viruses (barley/cereal yellow dwarf viruses [B/CYDV’s]: CYDV‐RPV, BYDV‐SGV, and BYDV‐PAV), either singly (purple circles) or jointly (orange squares). (a) Prevalence of RPV (i.e., proportion of the exposed hosts that became infected) suggests resource‐dependent antagonism (e.g., competition) within hosts. Prevalence of RPV is relatively unresponsive to nutrients when alone (if anything, increasing with N) but decreases steeply with N in co‐inoculations. (b) In contrast, SGV suggests facilitation. Prevalence of SGV is relatively unresponsive to nutrients when alone (if anything, decreasing with P) but increases with high P and low N in co‐inoculations. (c) Finally, prevalence of PAV does not differ between single or co‐inoculations and suggests weak or no interactions within hosts. Colored planes show fits of logistic regressions (statistics: Tables 1 & Table S3). Planes for single inoculations (purple) correspond to the heat maps for each virus alone (a–c in Figure 1), also shown in two dimensions (Figure S1 & Figure S3)

**FIGURE 3:**
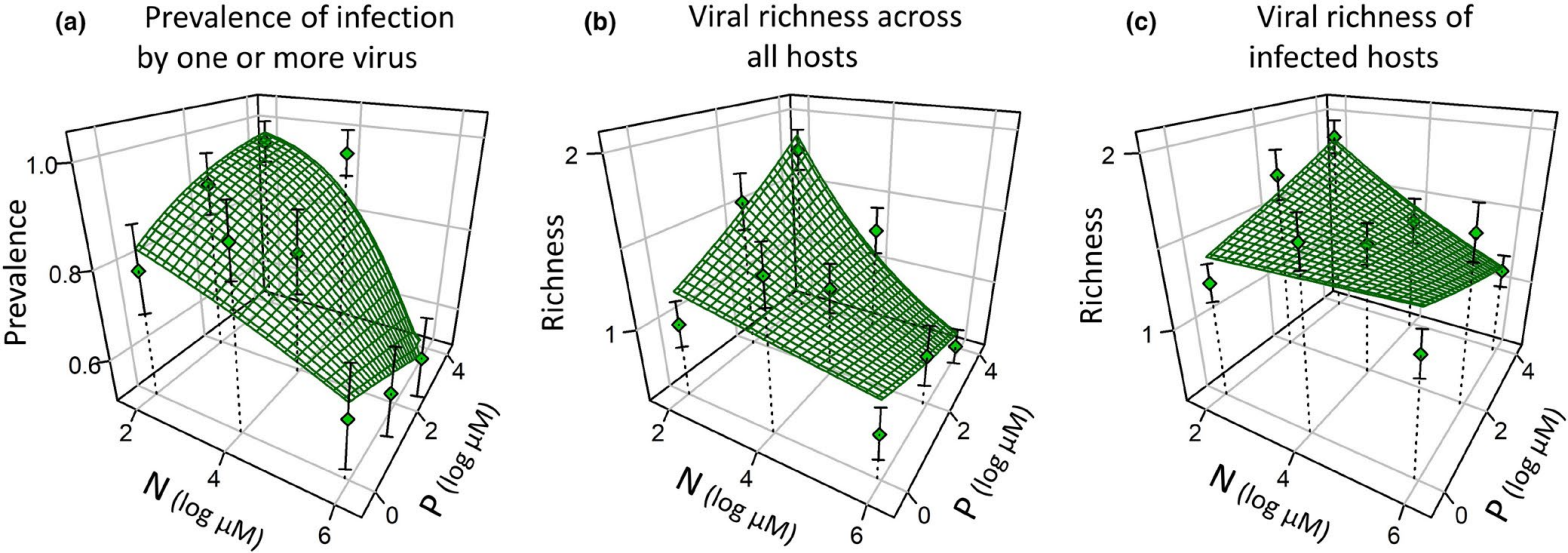
Viral richness peaks with the combination of low N and high P. Hosts (oats, *Avena* *sativa*) are grown under combinations of nitrogen and phosphorus supply (three levels each) and co‐inoculated with three viruses (barley/cereal yellow dwarf viruses [B/CYDV’s]: CYDV‐RPV, BYDV‐SGV, and BYDV‐PAV). (a) Overall prevalence among co‐inoculated hosts (i.e., proportion of exposed hosts that became infected by one or more viruses) increases with the combination of low N and high P (also shown as a heat map in Figure 1e). (b) Viral richness among all co‐inoculated hosts (average number of virus species per host) also increases with these resource conditions. (c) Among only the infected hosts, viral richness remains relatively constant across N and P. Planes show fits of linear models (statistics: Table 2). Error bars are standard errors. These responses of the virus community reflect the combination of each virus's responses in co‐inoculated hosts (orange planes in Figure 2). Specific combinations of viruses (e.g., RPV +SGV) shown in the appendix (Figure S2)

To visualize results, we graphically projected heat maps and smooth planes of infection risk in N×P space using the R package plot3D (Soetaert, 2014). First, we used heat maps to show infection risk for hosts exposed to each virus in isolation (Figure 1a‐c; statistics in Table S3) and composite risk in single inoculations with all viruses pooled together (Figure 1d; statistics in Table S4). This pooled model served as a qualitative prediction for the response of co‐inoculated hosts to N and P, assuming no interactions among viruses. We contrasted this prediction against the observed pattern of infection risk for co‐inoculated hosts; that is, the proportion of hosts infected by one or more virus (Figure 1e; statistics in Table 2). Next, we resolved the differences between these predicted and obs

erved patterns by showing how co‐inoculation altered the responses of each virus to N and P (Figure 2) as well as overall prevalence and realized richness of viruses in co‐inoculated hosts (Figure 3). We also collapsed our three‐dimensional results (infection prevalence in N×P space) onto two dimensions (Figure S1 in the appendix), tested responses of specific combinations of viruses to N and P (e.g., RPV and SGV together; Figure S2), and plotted all results along N:P ratios (Figure S3).

## 3 RESULTS

The responses of viruses to N and P were fundamentally different in isolation than in co‐inoculated hosts (Figure 1; Table 1). In single inoculations, RPV reached higher prevalence than SGV or PAV (*p* < .0001; Table S4), reflecting well‐known variation in transmission efficiency by the different aphid species (Rochow, 1969). We focus instead on relative effects of nutrients and co‐inoculation on infection risk. None of the viruses responded significantly to N or P in single inoculations (Figure 1a‐c; all *p >* .05; Table S3). The analysis with artificially inflated sample sizes showed that, if anything, RPV prevalence may have increased slightly with both N and P, and SGV prevalence may have decreased slightly with P (Table S3). When all single infections were pooled together (Figure 1d), infection prevalence was similarly insensitive to N and P (all *p* > .05; Table S4). However, infection risk for co‐inoculated hosts did increase with the combination of low N and high P (Figure 1e; N:P ratio: *p* = .0018; Table 2). This emergent pattern represented a qualitative divergence from the single inoculations, arising from resource‐dependent interactions within hosts.

Resource‐dependent antagonism and facilitation determined infection risk for co‐inoculated hosts (Box 1; Figure 2; Table 1). One virus—RPV—suffered from resource‐dependent antagonism, especially with increasing *N* (Figure 2a). At the lowest levels of N and P, RPV actually achieved a higher infection prevalence in co‐inoculations than single inoculations (R effect: *p* = .009; Table 1. However, this effect changed significantly with nitrogen (N×R interaction: *p* < .0001) and marginally with phosphorus (PxR interaction: *p* = .053). The post hoc models helped to interpret these interactions: In the single inoculations, RPV prevalence increased weakly with N and P (Table S3; both effects potentially significant with 20x replication), but in co‐inoculations it *decreased* steeply with *N* (N: *p* < .0001; Table S3). Thus, RPV suffered from antagonistic interactions among the viruses in co‐inoculated hosts (except, notably, at the lowest levels of N and P), and increasing N supply magnified this antagonistic effect (Figure 2a).

The other two viruses responded differently to N and P. SGV benefited from resource‐dependent facilitation (Figure 2b). In the crossed NxP model, infection prevalence of SGV differed significantly with interactions between nutrients (N×P: *p* =.018; Table 1) and between inoculated viral richness and P (P×R: *p* = .005). It also differed marginally with inoculated viral richness (R: *p* = .076) and its interaction with nitrogen (N×R: *p* = .082). The post hoc models and N:P ratio model helped to interpret these interactions. In single inoculations, SGV did not respond strongly to nutrients (Table S3). If anything, SGV prevalence may have decreased with P in single inoculations (potentially significant with 20× replication). However, in co‐inoculations, SGV prevalence *increased* with P (P: *p* < .001; Table S3) and this effect was significantly weaker with increasing *N* (N×P: *p* = .007). The N:P ratio model confirmed that prevalence increased with inoculated viral richness (R: *p* < .0001; Table 1) and that this effect declined at higher N:P ratios (N:P×R: *p* < .001). Graphically, prevalence of SGV clearly increases with co‐inoculation, especially with high P and low *N* (Figure 2b).

The third virus, PAV, generally reached low infection prevalence (under 30%) and experienced neither antagonism nor facilitation (Figure 2c). Prevalence of PAV increased with nitrogen (N: *p* = .019; Table 1), and the post hoc models showed that this effect was somewhat stronger in co‐inoculations (*p* = .018; Table S3) than single inoculations (*p* = .25). The crossed NxP PAV model confirmed that prevalence was unaffected by co‐inoculation (R: *p* =.93; Table 1). Thus, among the three viruses, we detected cases of resource‐dependent antagonism (RPV), resource‐dependent facilitation (SGV), and weak or no interactions within hosts (PAV).

Together, these interactions among viruses shaped patterns of infection risk and viral richness in co‐inoculated hosts (Figure 3; Table 2). For these hosts, the risk of infection by one or more viruses peaked at the combination of low N and high P (Crossed N×P model: N: *p* = .20, P: *p* = .063, N×P: *p* = .044; N:P ratio: *p* =.0018; Table 2). This result (Figure 1e & Figure 3a) reflects both the resource‐dependent outcome of antagonism for RPV (which was excluded with increasing N; Figure 2a) and the resource‐dependent facilitation of SGV (which was facilitated with increasing P; Figure 2b). Realized viral richness across all hosts (mean = 1.15 viruses per host) showed a very similar pattern and also peaked at the combination of low N and high P (Figure 3b; N:P ratio: *p* =.0038; Table 2). Thus, hosts that were exposed to multiple pathogens were more likely to become infected *and* more likely to contain multiple viruses with low N and high P. However, realized viral richness among only the infected co‐inoculated hosts (mean = 1.50 viruses per infected host) did not differ with N or P (Figure 3c; both *p* > .2). Thus, for hosts that became infected, resources and resource ratios did not shape within‐host viral richness. In other words, the supply of host resources was more influential in determining whether a host became infected at all, and less influential in setting an upper constraint on viral richness.

## 4 DISCUSSION

A growing body of literature seeks to link variation in infection risk to the resources of plant (Dordas, 2008; Veresoglou et al., 2013), animal (Becker et al., 2018; Hite et al., 2019), and human hosts (Prentice et al., 2008; Rohr et al., 2019). However, this literature largely fails to grapple with the reality that most hosts face communities of diverse pathogens. Importantly, mixed infections could fundamentally alter relationships between resources and disease (Budischak et al., 2015; Kendig et al., 2020; Lacroix et al., 2014; Lange et al., 2014; Strauss et al., 2019; Wale et al., 2017). Our experimental work demonstrated that the responses of three viruses to N and P in isolation qualitatively misguided predictions of risk for hosts that were inoculated with all three viruses together. The single virus responses suggested that risk for co‐inoculated hosts would not vary strongly with nutrients. Instead, both infection risk and viral richness increased with the combination of low N and high P. This outcome emerged from both resource‐dependent antagonism and resource‐dependent facilitation within hosts. RPV was excluded with increasing N, whereas SGV was facilitated with decreasing N:P. Interactions such as these are likely widespread across viruses (Lacroix et al., 2014) and other pathogens (Budischak et al., 2015) that infect plants (Abdullah et al., 2017), animals (Wale et al., 2017), and humans (Corbett et al., 2003).

Predicting infection risk for hosts remains a central challenge in disease ecology. It is certainly tempting to use environmental variables, such as resource availability, to predict the infection risk for hosts that occur across variable environments (Schatz et al., 2017). Such projections play a valuable role in predicting disease emergence and spread in a changing world and could be especially important in the context of plant disease in sustainable agriculture (Dordas, 2008; Huber & Haneklaus, 2007; Mur et al., 2017). Yet such projections could be misleading if they are parameterized for single pathogens and fail to acknowledge the interactions within hosts that can arise in more diverse and realistic pathogen communities (DaPalma et al., 2010; Pedersen & Fenton, 2007). Here, the responses of each virus to nitrogen and phosphorus in isolation qualitatively misguided expectations of risk for co‐inoculated hosts. Based on the single inoculations, risk for co‐inoculated hosts seemed likely to remain relatively constant across N and P. Instead, both the risk of infection by one or more virus and viral richness in co‐inoculated hosts increased with the combination of low N and high P. These results are broadly consistent with field patterns, where hosts were exposed to natural communities of B/CYDVs, and infection prevalence and viral richness increased under P but not N fertilization (Seabloom et al., 2013). Thus, in order to generate accurate projections of disease risk from environmental variables, our results suggest the need to combine insights from field experiments where hosts are exposed to natural pathogen communities (e.g., Seabloom et al., 2013) and laboratory experiments that assess the effects of multiple interacting pathogens under varied environmental conditions (e.g., Budischak et al., 2015; Kendig et al., 2020; Lacroix et al., 2014).

Resource‐dependent antagonism among viruses inhibited infection by RPV at higher levels of nitrogen. The nutritional environment of hosts has long been proposed as a factor mediating interactions among pathogens within hosts (Hite et al., 2019; Smith, 2007; Smith & Holt, 1996). Pathogens often compete for shared resources (Griffiths et al., 2014), and models show that these dynamics can structure the diversity of pathogen communities both within and among hosts (Strauss et al., 2019). Importantly, identifying resource‐dependent competition among pathogens can suggest clinical strategies to slow the evolution of drug resistance (Wale et al., 2017) and the evolution of virulence (Pulkkinen et al., 2018). Here, RPV reached relatively high infection prevalence when alone (mean 69%), but was excluded at higher rates of nitrogen supply in co‐inoculations. The interactions inside the host that drove this pattern remain unclear. Other experiments found that the titer of RPV was lower in hosts that were coinfected with PAV (Lacroix et al., 2017) and that PAV excluded RPV under conditions of low N and low P (Lacroix et al., 2014). However, presence of PAV *increased* titer of RPV in successful infections when N supply was high (Kendig et al., 2020). Differences in these results suggest that 1) interactions could switch from antagonistic at early stages of infection to synergistic later on, potentially when the plant immune system is more active, and 2) that RPV is a poor competitor in the initial stages of infection, but that the resource environment that renders it most susceptible to exclusion may depend on the diversity and identity of other viruses (i.e., higher‐order interactions). Greater mechanistic understanding of these interactions could lead to better a priori predictions of disease dynamics along nutrient gradients. Importantly, these results show that under certain environmental conditions, risk of infection by multiple pathogens may be less severe than expected, due to increasingly antagonistic interactions within hosts.

In contrast, resource‐dependent facilitation increased infection prevalence of SGV. Thus, resource‐dependent projections of disease risk based on single pathogen responses could also *underestimate* risk of infection by diverse pathogen communities. Facilitation can arise when one pathogen attacks the host's immune function and enables infection by another. As a classic example, infection by HIV increases infection risk of tuberculosis in humans (Corbett et al., 2003). Furthermore, it is becoming increasingly clear that host resources can mediate facilitation among pathogens (van Lettow et al., 2003). For instance, protein limitation in mice altered immune‐mediated facilitation among helminths and intracellular parasites (Budischak et al., 2015). In the current study, SGV reached very low infection prevalence when alone (mean 10%), but reached much higher prevalence in co‐inoculation, especially with high P and low N supply (up to 85%). One explanation invokes heterologous encapsidation, where one virus hijacks the capsid protein of others (Wen & Lister, 1991). An alternative explanation invokes RNA silencing defenses of the host (Waterhouse et al., 2001), inhibition of these defenses by RPV and/or PAV (Liu et al., 2012), and different resource requirements for these defenses of hosts (e.g., stronger with nitrogen (Mur et al., 2017)) and counter‐defenses of the viruses (e.g., stronger with phosphorus (Clasen & Elser, 2007)). In general, these patterns of facilitation could warn of alarming increases in disease risk with combinations of resources that fuel pathogen infectivity and inhibit host defense.

Existing theoretical frameworks from community ecology could help disease ecologists grapple with these complex relationships between host resources and infection risk from diverse pathogen communities (Rynkiewicz et al., 2015; Seabloom et al., 2015). One obvious place to start is resource ratio (R*) theory (Smith & Holt, 1996; Tilman, 1977), with extensions to include apparent competition (Holt, 1977) mediated by host immune function (i.e., cross‐protection among pathogens (Wen et al., 1991)). Two major caveats are that immune function can also rely directly on resources in a sense that predators do not (Cressler et al., 2014; Smith & Holt, 1996) and that pathogens can inhibit the immune function of hosts (Budischak et al., 2015; Corbett et al., 2003; Liu et al., 2012). Moreover, infection risk is not determined exclusively by dynamics operating within hosts: It also depends on exposure to pathogens. For vector‐borne pathogens, inherent differences in vector ecology therefore play a large role in determining risk of infection by different pathogens (Kendig et al., 2017; Seabloom et al., 2013; Strauss et al., 2020). From a theoretical perspective, R* theory can be nested within a metacommunity framework—where each patch is a host—to link resource ratios to disease dynamics both within and among hosts (Borer et al., 2016; Strauss et al., 2019). This approach could also accommodate priority effects among pathogens (Halliday et al., 2017) and with host immune function (Cressler et al., 2014). Importantly, in this study system, N and P can also shape aphid demography (Zehnder & Hunter, 2009), potentially altering risk of infection by amplifying vector populations (Strauss et al., 2020). Thus, expansions of R* theory that are tailored to disease must allow resource‐dependent antagonism, resource‐dependent facilitation, and potentially resource‐dependent transmission/dispersal. Such expansions promise to generate a new range of dynamics that may advance our understanding of disease in a wide range of hosts.

We showed here that infection risk for hosts depends on resource‐dependent interactions inside hosts ranging from antagonism to facilitation. If we continue to largely ignore these divergent outcomes that can occur among pathogens in response to the same environmental changes, then we risk being entirely wrong in our projections of infection risk across environmental gradients such as resource availability. It is becoming increasingly urgent to understand these linkages among resources, pathogen diversity, and disease, as anthropogenic forces continue to alter the availability of nutrient resources to plant, animal, and human hosts. Expanded resource competition and metacommunity theory, tailored to host‐parasite biology, could promote a more mechanistic understanding of these linkages. If different pathogens—or combinations of pathogens—pose greater risk to hosts than others, then knowledge of the resource‐dependent interactions among these pathogens could provide essential information for medical and agricultural treatment strategies, as well as predicting infection risk, disease spread, and host morbidity and mortality.

## CONFLICT OF INTEREST

We declare no competing interests.

## AUTHOR CONTRIBUTION

**Alexander Strauss:** Conceptualization (equal); Formal analysis (lead); Funding acquisition (supporting); Investigation (equal); Project administration (equal); Writing‐original draft (lead); Writing‐review & editing (equal). **Lucas** **Bowerman:** Investigation (equal); Writing‐review & editing (equal). **Anita** **Porath‐Krause:** Investigation (equal); Project administration (equal); Writing‐review & editing (equal). **Eric** **Seabloom:** Conceptualization (equal); Funding acquisition (equal); Writing‐review & editing (equal). **Elizabeth Borer:** Conceptualization (equal); Funding acquisition (equal); Writing‐review & editing (equal).

## Supporting information

Appendix

## Data Availability

All data and code has been made publicly available on Dryad: DOI https://doi.org/10.5061/dryad.8931zcrr2
