# Supplementary material for: Mixed infection, risk projection, and misdirection: Interactions among pathogens alter links between host resources and disease
Source: Ecol Evol. 2021 Jun 22;11(14):9599–609. doi: 10.1002/ece3.7781 (PMC8293790; doi:10.1002/ece3.7781)
Supplement: Supplementary file 1 — Appendix [file ECE3-11-9599-s001.docx]

**Appendix:**

**Mixed infection, risk projection and misdirection: Interactions among pathogens alter links between host resources and disease**

**APPENDIX**

In this appendix we present additional methodological details and supplementary results. First, we describe maintenance of aphid vector and virus populations in the lab. Then, we describe recipes of the nutrient solutions used to create crossed gradients of nitrogen and phosphorus supply in the experiment (Table S1). Then, we provide details describing the molecular analyses used to diagnose infections in the experimental plants (Table S2). Next, we report the additional statistical analyses that split the data for each virus into single versus co-inoculations, to better interpret the effects of nutrients and their ratios (Table S3). We also tested whether our unbalanced experimental design, with greater replication for the co-inoculated than singly-inoculated hosts, altered the interpretation of our results (see footnote in Table S3). We also report statistics of the models that pooled all single inoculations together (Table S4). Then, we collapse the three-dimensional NxP space as presented in the main text (Fig. 2) to show two-dimensional infection prevalence across the gradient of N (with P levels as contours) and the gradient of P (with N levels as contours; Fig. S1). We also graphically (Fig. S2) and statistically (Table S5) show how specific combinations of viruses (e.g., RPV and SGV together; all three viruses together) responded to N and P. Finally, we show the prevalence of both single and multiple infections across gradients of N:P ratios, instead of cross gradients of N and P (Fig. S3).

**Maintenance of vector and virus populations**

All three viruses (CYDV-RPV, BYDV-SGV, and BYDV-PAV) were originally isolated by the laboratory of Dr. Stewart Gray from cereal crops in New York state (Cornell University, Ithaca, NY). Since obtaining each virus, our lab group has continuously maintained their strains by transferring ~25 viruliferous aphids to new cohorts of cultivated oats (*A. sativa*) every ~3 weeks. These plants are grown in Sunshine MVP potting soil (Sun Gro Horticulture) in 15 x 15 cm pots and watered twice per week with tap water. These plants are routinely confirmed to be infected by each virus, following standard protocols (Gray 2008). During this experiment, we maintained these long-term cultures of plants, aphids, and viruses in a separate room from the experiment but under similar conditions (23°C; 16:8 light:dark; 2x 40 W cool white fluorescent bulbs).

**Nutrient solutions**

We watered plants in the experiment with modified Hoagland’s nutrient solutions (Hoagland & Arnon 1950). Concentrations of both nitrogen (as ammonium nitrate) and phosphorus (as monopotassium phosphate) corresponded to 0.1%, 0.7%, or 5% dilutions of the original recipe. Concentrations of all micronutrients and macronutrients are listed below (Table S1).

**Table S1.** *Nutrient solutions.*

| Compound | Formula | Concentration (µM) |
| --- | --- | --- |
| potassium sulfate | K_2_SO_4_ | 1250 |
| magnesium sulfate | MgSO_4_.7H_2_O | 1000 |
| calcium sulfate | CaSO_4_·2H_2_O | 2000 |
| potassium chloride | KCl | 25 |
| boric acid | H_3_BO_3_ | 12.5 |
| magnesium sulfate | MnSO_4_·H_2_O | 1 |
| zinc sulfate | ZnSO_4_·7H_2_O | 1 |
| copper sulfate | CuSO_4_·5H_2_O | 0.25 |
| molybdic acid | H_2_MoO_4_·(H_2_O) | 0.25 |
| ferric sodium EDTA | NaFeEDDHA (6% Fe) | 10 |
| monopotassium phosphate | KH_2_PO_4_ | 1, 7, or 50* |
| ammonium nitrate | NH_4_NO_3_ | 7.5, 52.5, or 375* |

** depending on nutrient treatment*

**Diagnosing infections from plant tissues**

We diagnosed infections in plant hosts following standard laboratory procedures (e.g., Lacroix *et al.* 2014). In summary, we flash-froze plant tissues, extracting total RNA with TRIzol® Reagent (Invitrogen^TM^) and chloroform (since B/CYDV’s are single-stranded RNA viruses), synthesized cDNA with generic primers, amplified virus cDNA with primers specific to each virus species (Table S2), and used gel electrophoresis to visually diagnose whether plants were infected.

We extracted total RNA following a standard laboratory protocol. Immediately after sampling, we cut 0.04-0.07g of tissue from each plant (from the newest leaf, if possible) and flash-froze it in liquid nitrogen. Later, we cut these frozen tissue samples into 1-2 mm pieces, added them to microcentifuge tubes containing 500 μl TRIzol®, and pulverized them with steel BBs in a bead beater at 10 second intervals until fully homogenized (Mini-Beadbeater-16 Biospec Products). Then we added 100 μl chloroform to the tubes, mixed by inverting (15 s), and cold-centrifuged (4 C, 7,000 g, 15 min). We transferred the aqueous phases to new tubes containing 100 μl isopropanol, mixed by inverting, and cold-centrifuged again (7,000 g, 10 min). Next, we discarded the supernatant, added 1 ml 75% ethanol, briefly vortexed our samples, cold-centrifuged for a third time (4 C, 7,000 g, 5 min), and discarded the supernatant. Finally, we allowed the pellets containing RNA to dry (minimum 30 min) before dissolving the pelleted RNA in 20 μl RNase-free water and freezing these total RNA samples for future use (-20 C).

We synthesized complementary DNA (cDNA) from the total RNA samples using reverse transcription polymerase chain reactions (RT-PCR). We mixed 4.5 μl of RNA solution from each sample and 0.5 μl of random hexamers (1ug/ul) and preheated these mixtures (70 C, 5 min) in a thermocycler (S1000^TM^ Thermal Cycler [Bio-Rad]). Each RT-PCR reaction (20 ul) contained 5 μl of this random hexamer/RNA mixture, 4 μl 5x Reaction Buffer (ImProm-II^TM^ Reverse Transcriptase [Promega]), 1.2 μl MgCl_2_ (25mM), 1 μl dNTPs (10mM), 0.5 μl Recombinant RNasin® Ribonuclease Inhibitor (Promega; 40U/ul), 1 μl (ImProm-II^TM^ Reverse Transcriptase [Promega]), 7.3 μl RNase free water, and 0.034 μl T4 Gene 32 Protein (New England BioLabs). Thermocycler conditions for cDNA synthesis were 5 min at 25 C, 60 min at 45 C, and finally 15 min at 70 C.

Next, we amplified viral cDNA with virus-specific primers and thermocycler conditions via PCR. For co-inoculated hosts, separate reactions amplified potential cDNA of each virus. Each reaction (20 ul) included 2 μl 10x buffer, 2.8 μl MgCl_2_ (25mM), 10.4 μl nanopure water, 0.8 μl each forward and reverse primers (10 uM), 0.8 μl dNTPs (10 mM), 0.4 μl HotStarTaq® DNA Polymerase (Qiagen), and 0.068 μl T4 Gene 32 Protein (New England BioLabs). Finally, we used gel electrophoresis to visually diagnose infections. We loaded the amplified DNA samples into 2.0% gel (UltraPure Agarose-1000, Thermo Fisher Scientific) mixed with SYBR Safe DNA Gel Stain (Invitrogen^TM^) and visualized with Gel Doc^TM^ EZ Imager (Bio Rad).

**Table S2.** *Virus-specific primers & thermocycler conditions*

| Virus | Forward Primer | Reverse Primer | Thermocycler conditions |
| --- | --- | --- | --- |
| CYDV-RPV | RPV 3262F:  5' - ATG TTG TAC CGC TTG ATC CAC - 3' | RPV 3859R:  5' - CTG CGT TCT GAC AGC AGG - 3' | Initial heating phase (95 C, 15 min); amplification phase (95 C [30 s], 59 C [30 s], and 72 C [60 s] for 19 cycles, and then for 20 cycles (95 C [30 s], 55 C [30s], 72 C [60 s]), and a final extension of 72 C [10 min]. |
| BYDV-SGV | SGV L2:  5’ – ACC AGA TCT TAG CCG GGT TT -3’ | SGV R2:  5’ – CTG GAC GTC GAC CAT TTC TT – 3’ | Initial heating phase (95 C, 15 min); step-down phase (95 C [30 s], 59 C [30 s], and 72 C [30 s] with subsequent annealing iterations reduced from 59 C to 54 C in 1 C increments); and then 31 cycles at (95 C [30 s], 54 C [30 s], and 72 C [30 s]) and a final extension of 72 C [10 min]. |
| BYDV-PAV | PAV 3082F:  5' - CCT TAA AGC CAA CTC TTC CG - 3' | PAV 3288R:  5’ - TAG CTA GCC AGG GCT GAT T - 3' | Initial heating phase (95 C, 15 min); step-down phase (95 C [30 s], 59 C [30 s], and 72 C [30 s] with subsequent annealing iterations reduced from 59 C to 54 C in 1 C increments); and then 31 cycles at (95 C [30 s], 54 C [30 s], and 72 C [30 s]) and a final extension of 72 C [10 min]. |

**Table S3.** *Statistical tests separately for single inoculations and co-inoculations.* We omitted interaction terms if they were not significant to avoid overfitting the models. We also tested which of the results from single inoculation could have become significant with greater replication. For these analyses, we duplicated each observed result, yielding a sample size of 20x, consistent with the con-inoculations. Here we note which *p*-values became significant following this artificial inflation. Note that these results (RPV *increasing* with N and P in single inoculations; SGV *decreasing* with P in single inoculations) are opposite in direction than in the co-inoculations. Therefore, if anything, our unbalanced design underestimated the differences between single and inoculations and the strength of interactions among pathogens.

| **Single Inoculations** | response: RPV  (Figs. 1A & 2A) | | | response: SGV  (Figs. 1B & 2B) | | | response: PAV  (Figs. 1C & 2C) | | |
| --- | --- | --- | --- | --- | --- | --- | --- | --- | --- |
| Crossed N x P | est. | s.e. | *p* value | est. | s.e. | *p* value | est. | s.e. | *p* value |
| intercept* | -0.13 | 0.45 | 0.77 | -0.80 | 0.49 | 0.10 | -2.20 | 0.66 | <0.001 |
| N^†^ | 0.29 | 0.15 | 0.052**^‡^** | -0.05 | 0.17 | 0.75 | 0.22 | 0.19 | 0.25 |
| P^†^ | 0.21 | 0.15 | 0.16**^‡^** | -0.28 | 0.17 | 0.11**^‡^** | -0.01 | 0.19 | 0.96 |
| N:P ratio | est. | s.e. | *p* value | est. | s.e. | *p* value | est. | s.e. | *p* value |
| intercept | 0.72 | 0.30 | 0.017 | -1.62 | 0.38 | <0.0001 | -2.00 | 0.44 | <0.0001 |
| N:P^†^ | 0.04 | 0.10 | 0.69 | 0.11 | 0.12 | 0.36 | 0.11 | 0.13 | 0.40 |
| **Co-Inoculations** | response: RPV  (Fig. 2A) | | | response: SGV  (Fig. 2B) | | | response: PAV  (Fig. 2C) | | |
| Crossed N x P | est. | s.e. | *p* value | est. | s.e. | *p* value | est. | s.e. | *p* value |
| intercept* | 1.35 | 0.35 | <0.001 | -0.29 | 0.40 | 0.47 | -3.02 | 0.76 | <0.001 |
| N^†^ | **-0.54** | **0.11** | **<0.0001** | -0.11 | 0.16 | 0.51 | **0.58** | **0.24** | **0.018** |
| P^†^ | -0.03 | 0.17 | 0.86 | **0.68** | **0.19** | **<0.001** | 0.30 | 0.26 | 0.25 |
| N x P |  |  |  | **-0.20** | **0.07** | **0.007** | -0.14 | 0.09 | 0.12 |
| N:P ratio | est. | s.e. | *p* value | est. | s.e. | *p* value | est. | s.e. | *p* value |
| intercept | 0.41 | 0.21 | 0.054 | 0.76 | 0.23 | 0.001 | -2.09 | 0.33 | <0.0001 |
| N:P^†^ | **-0.19** | **0.07** | **0.0067** | **-0.37** | **0.08** | **<0.0001** | 0.16 | 0.10 | 0.092 |

* Intercept in Crossed N x P models is log odds at lowest levels of N and P in the experiment

† N, P, and N:P ratio are log transformed to reduce statistical leverage

‡ Effect became statistically significant with artificial inflation of sample size from 10x to 20x

**Table S4.** *Statistical tests for all single-inoculations pooled together*

|  | With ‘virus species’ as factor | | | Without ‘virus species’ as factor  (Fig. 1D) | | |
| --- | --- | --- | --- | --- | --- | --- |
| Crossed N x P | est. | s.e. | *p* value | est. | s.e. | *p* value |
| intercept* | 0.50 | 0.34 | 0.14 | -0.86 | 0.26 | 0.001 |
| N^†^ | 0.16 | 0.09 | 0.095 | 0.12 | 0.08 | 0.14 |
| P^†^ | -0.00 | 0.09 | 0.98 | -0.00 | 0.08 | 0.96 |
| Virus: PAV | **-2.58** | **0.38** | **<0.0001** |  |  |  |
| Virus: SGV | **-2.21** | **0.35** | **<0.0001** |  |  |  |
| N:P ratio | est. | s.e. | *p* value | est. | s.e. | *p* value |
| intercept | 0.64 | 0.26 | 0.014 | -0.76 | 0.18 | <0.0001 |
| N:P^†^ | 0.08 | 0.07 | 0.23 | 0.06 | 0.06 | 0.28 |
| Virus: PAV | **-2.56** | **0.38** | **<0.0001** |  |  |  |
| Virus: SGV | **-2.19** | **0.35** | **<0.0001** |  |  |  |
|  |  |  |  |  |  |  |

* Intercept in Crossed N x P models is log odds at lowest levels of N and P in the experiment

† N, P, and N:P ratio are log transformed to reduce statistical leverage

**Infection prevalence compressed to two-dimensional space
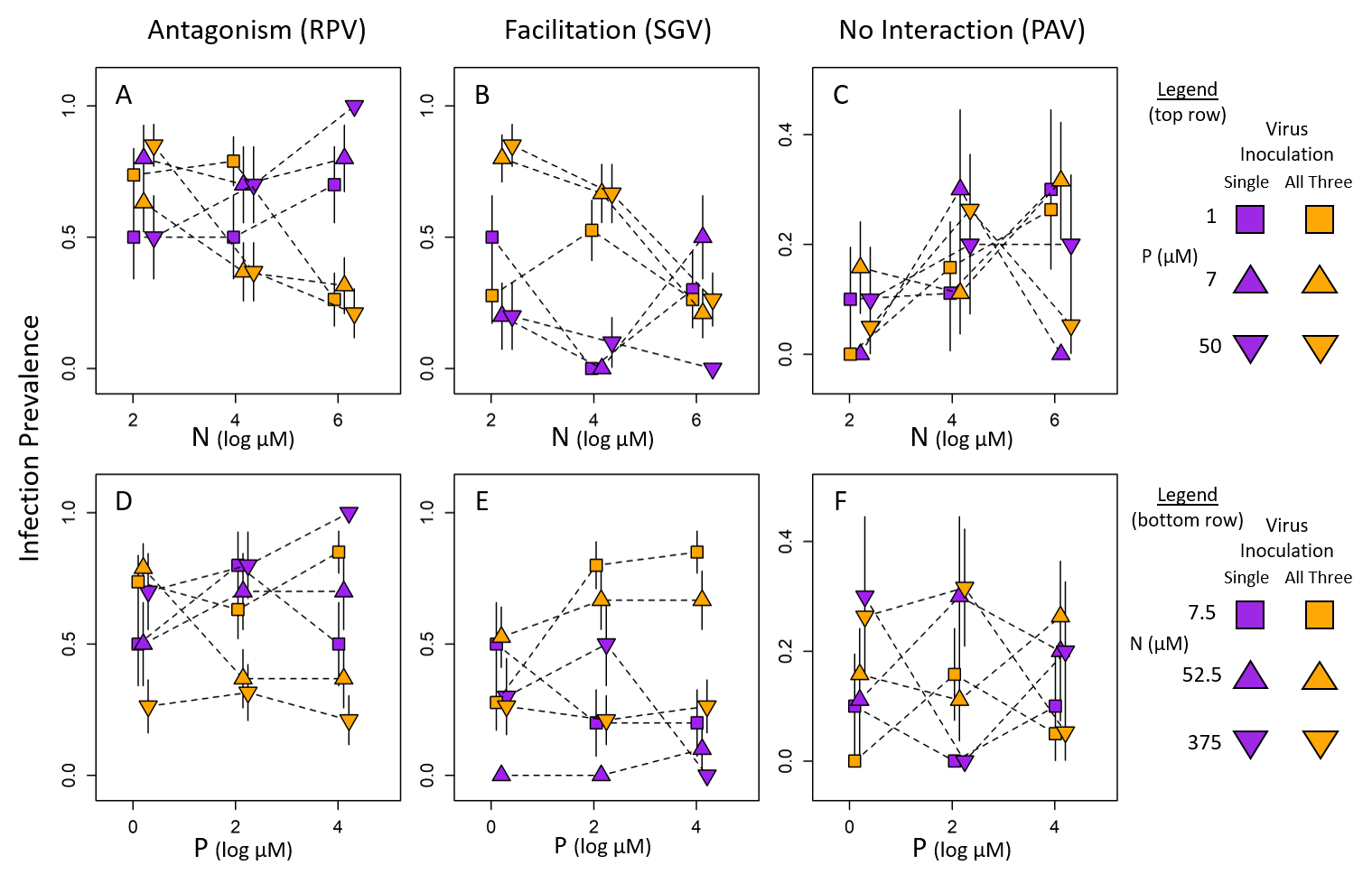
**

**Figure S1.** *Infection prevalence compressed to two-dimensional space. Data are identical to those presented in three-dimensional space in the main text (infection prevalence in NxP space; Fig. 2).* Hosts (oats, *Avena sativa*) are grown under combinations of nitrogen and phosphorus supply (three levels each) and inoculated with three viruses (barley/cereal yellow dwarf viruses [B/CYDV’s]: CYDV-RPV, BYDV-SGV, and BYDV-PAV [columns]), either singly (purple) or all together (orange). Top row: Infection prevalence (i.e., proportion of exposed hosts that became infected) across the gradient of N, with P levels as contours (unique shapes; connected by dashed lines). Bottom row: Infection prevalence across the gradient of P, with N levels as contours (unique shapes; connected by dashed lines). **A,D)** Prevalence of RPV suggests resource-dependent antagonism (e.g., competition) within hosts. Prevalence of RPV increases weakly with N when alone but decreases steeply with N in co-inoculations. **B,E)** In contrast, SGV suggests facilitation. Prevalence of SGV decreases slightly with P when alone but increases steeply with P in co-inoculations. **C,F)** Finally, PAV suggests no interactions within hosts: Prevalence of PAV does not differ between single or co-inoculations. Error bars are standard errors; statistics presented in Table 1 of the main text.

**Specific types of coinfections**


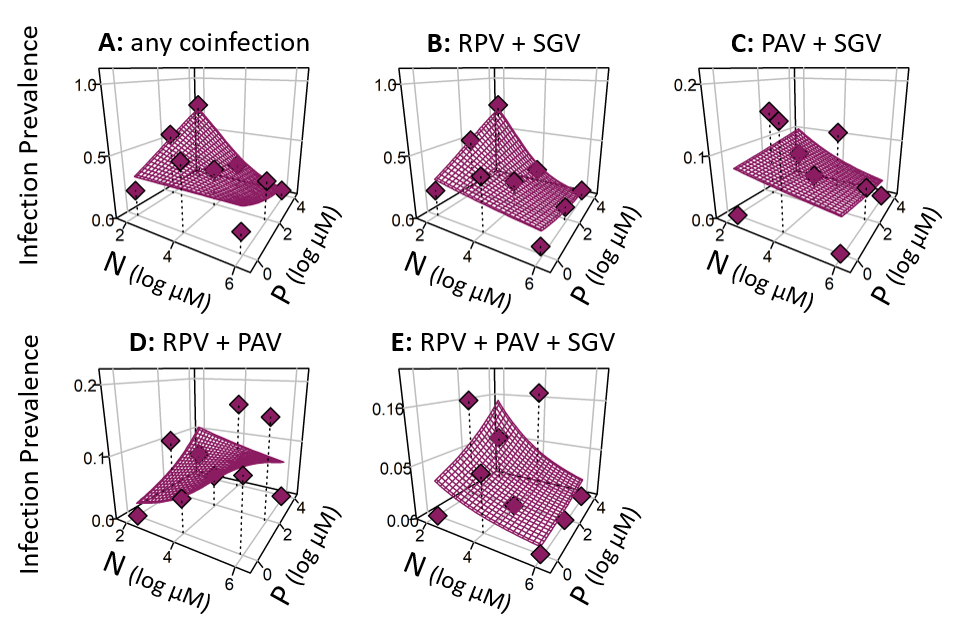


**Figure S2.** *Prevalence of coinfections across gradients of nitrogen and phosphorus.* Hosts (oats, *Avena sativa*) are grown under combinations of nitrogen and phosphorus and inoculated with three viruses together (RPV, PAV, SGV). Prevalence of **A)** any combination of two or more viruses and **B)** specifically RPV and SGV together is highest with the combination of low N and high P. Neither N nor P alter the prevalence of coinfections of **C)** PAV and SGV together, **D)** RPV and PAV together, or **E)** all three viruses together. Colored planes show fits of logistic regression models (statistics summarized in Table S5).

**Table S5.** Effects of nitrogen (N) and phosphorus (P) on the prevalence of specific combinations of viruses (graphically in Fig. S2). Non-significant interaction terms were omitted to avoid overfitting models.

|  | any coinfection  (Fig. S2A) | | | RPV + SGV  (Fig. S2B) | | | PAV + SGV  (Fig. S2C) | | |
| --- | --- | --- | --- | --- | --- | --- | --- | --- | --- |
| Crossed N x P | est. | s.e. | *p* value | est. | s.e. | *p* value | est. | s.e. | *p* value |
| intercept* | -0.73 | 0.40 | 0.069 | -0.84 | 0.42 | 0.045 | -2.30 | 0.58 | <0.0001 |
| N^†^ | 0.01 | 0.16 | 0.97 | -0.21 | 0.19 | 0.28 | -0.22 | 0.20 | 0.28 |
| P^†^ | **0.42** | **0.16** | **0.010** | **0.44** | **0.17** | **0.0085** | -0.01 | 0.20 | 0.97 |
| N x P | **-0.23** | **0.07** | **0.0024** | **-0.29** | **0.09** | **0.0089** |  |  |  |
| N:P ratio | est. | s.e. | *p* value | est. | s.e. | *p* value | est. | s.e. | *p* value |
| intercept | -0.23 | 0.21 | 0.26 | -0.37 | 0.21 | 0.078 | -2.51 | 0.38 | <0.0001 |
| N:P^†^ | **-0.22** | **0.08** | **0.0031** | **-0.37** | **0.09** | **<0.0001** | -0.10 | 0.14 | 0.45 |
|  | RPV + PAV  (Fig. S2D) | | | RPV + PAV + SGV  (Fig. S2E) | | |  | | |
| Crossed N x P | est. | s.e. | *p* value | est. | s.e. | *p* value |  |  |  |
| intercept* | -2.92 | 0.63 | <0.0001 | -3.33 | 0.89 | <0.001 |  |  |  |
| N^†^ | 0.14 | 0.18 | 0.45 | -0.43 | 0.31 | 0.16 |  |  |  |
| P^†^ | 0.06 | 0.18 | 0.74 | 0.27 | 0.28 | 0.33 |  |  |  |
| N x P |  |  |  |  |  |  |  |  |  |
| N:P ratio |  |  |  |  |  |  |  |  |  |
| intercept | -2.59 | 0.40 | <0.0001 | -2.91 | 0.43 | <0.0001 |  |  |  |
| N:P^†^ | 0.04 | 0.13 | 0.76 | -0.35 | 0.21 | 0.092 |  |  |  |

* Intercept in Crossed N x P models is log odds at lowest levels of N and P in the experiment

† N, P, and N:P ratio are log transformed to reduce statistical leverage

**
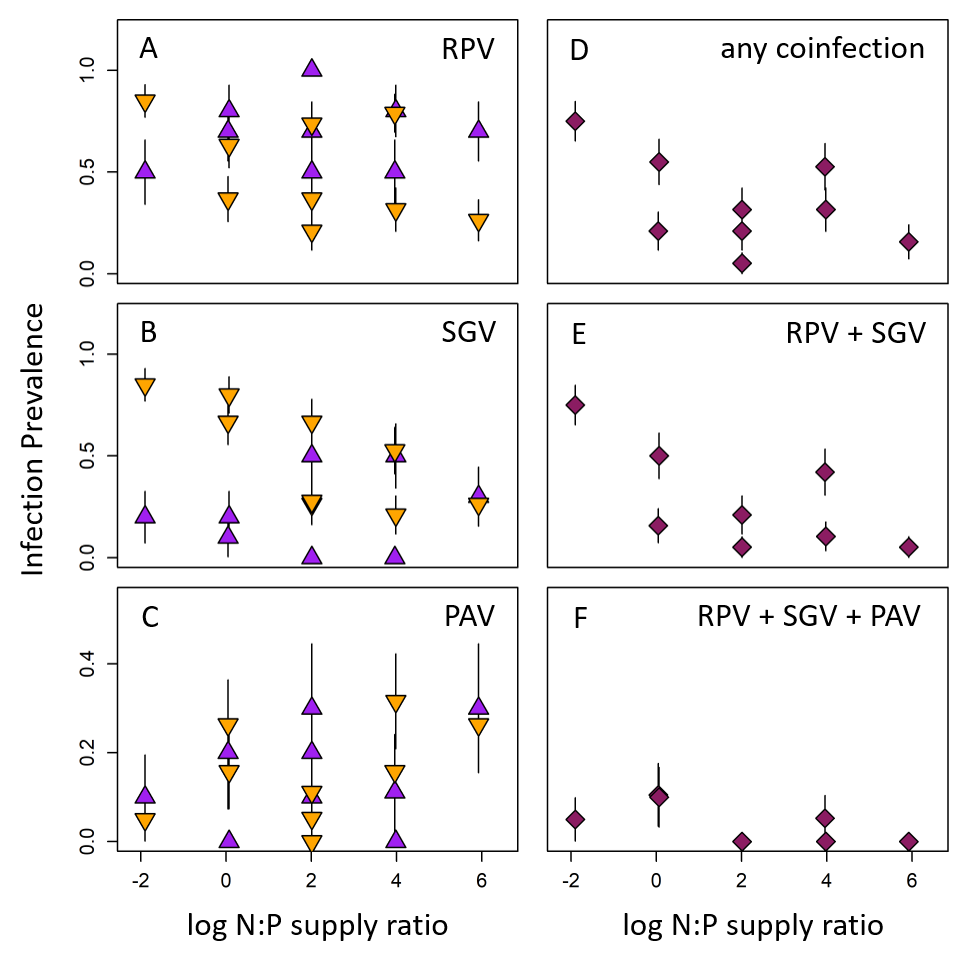
**

**N:P ratios as predictors**

**Figure S3.** *Prevalence of single and coinfections across a gradient of N:P ratios.* Hosts (oats, *Avena sativa*) are grown under combinations of nitrogen and phosphorus and inoculated with three viruses together (RPV, PAV, SGV). Left column: Infection prevalence of each virus alone (downward orange triangles) and in co-inoculations (upward purple triangles), including A) RPV, B) SGV, and C) PAV; analogous to Fig. 2 in the main text but collapsing 3D NxP space into a single axis of N:P ratios. Right column: The proportion of hosts infected by combinations of viruses, including D) any combination, E) specifically RPV + SGV, or F) all three viruses together; analogous to Fig. S2 but collapsing 3D NxP space into a single axis of N:P ratios. Error bars are standard errors.

**REFERENCES**

1.

Gray, S.M. (2008). Aphid transmission of plant viruses. *Current Protocols in Microbiology*, 10, 16B.11.11-16B.11.10.

2.

Hoagland, D.R. & Arnon, D.I. (1950). The water-culture method for growing plants without soil. *Circular. California Agricultural Experiment Station*, 347, 32 pp.

3.

Lacroix, C., Seabloom, E.W. & Borer, E.T. (2014). Environmental nutrient supply alters prevalence and weakens competitive interactions among coinfecting viruses. *New Phytol.*, 204, 424-433.
